# Multi-laboratory evaluations of XMRV nucleic acid detection assays

**DOI:** 10.1186/1742-4690-8-S1-A231

**Published:** 2011-06-06

**Authors:** Graham Simmons, John M Coffin, Indira K Hewlett, Shyh-Ching Lo, Judy A Mikovits, William M Switzer, Jeffrey M Linnen, Francis Ruscetti, Simone A Glynn, Michael P Busch

**Affiliations:** 1 Blood Systems Research Institute, San Francisco, CA 94118, USA; 2 Department of Laboratory Medicine, University of California, San Francisco, San Francisco, CA, 94118, USA; 3 National Cancer Institute-Frederick, MD, USA; 4 Department of Molecular Biology and Microbiology, Tufts University, Boston, MA, 02111, USA; 5 Office of Blood Research and Review, FDA, Bethesda, MD, 20892, USA; 6 Division of Cellular and Gene Therapies and Division of Human Tissues, FDA, Bethesda, MD, 20892, USA; 7 Whittemore Peterson Institute, Reno, Nevada, 89557, USA; 8 University of Nevada, Reno, NV, 89557, USA; 9 Laboratory Branch, Division of HIV/AIDS Prevention, CDC, Atlanta, GA, 30333, USA; 10 Gen-Probe Incorporated, San Diego, CA, 92121, USA; 11 Laboratory of Experimental Immunology, National Cancer Institute- Frederick, Frederick, MD, 21701, USA; 12 Transfusion Medicine and Cellular Therapeutics Branch, NHLBI, Bethesda, MD, 20892, USA

## 

The Blood XMRV Scientific Research Working Group was formed to facilitate collaborative studies into the impact of XMRV in blood donors. Studies will evaluate XMRV detection assays in terms of sensitivity, specificity and reproducibility; assess performance on specimens represented in existing blood donor repositories, and determine the prevalence of XMRV in donors. Phase I utilized analytical performance panels spiked with XMRV infected cells or virus. These panels were tested in a blinded fashion using XMRV nucleic acid testing (NAT) assays developed by six participating laboratories, with all laboratories determined to have sensitive NAT assays. Phase II represented pilot studies to compare XMRV detection using PBMCs, WB and plasma derived from individuals identified as XMRV viremic and antibody positive in previous studies. An unblinded pilot study resulted in two laboratories detecting MLV-like sequences in the plasma, but not PBMCs or WB, from all four subjects. A third laboratory detected no viral sequences. A blinded pilot study using the same four subjects and two validated negatives was less conclusive, with 3/4 laboratories detecting no viral sequences with any of the samples. A FACS-based serological assay detected antibodies in 3/4 XMRV-positive individuals, but also in 1/2 negatives. Seroreactivity to XMRV was not observed in plasma samples by Western blot. Phase III involves further evaluation of the clinical sensitivity and specificity of candidate assays by using a blinded panel of 35 pedigreed positives, together with negatives and controls. Results are expected soon. Phase IV will test a blinded panel of 300 blood donor samples.

